# Supplementary figures and images for: Single versus repeated heat stress in wheat: What are the consequences in different developmental phases?
Source: PLoS One. 2021 May 25;16(5):e0252070. doi: 10.1371/journal.pone.0252070 (PMC8148339; doi:10.1371/journal.pone.0252070)

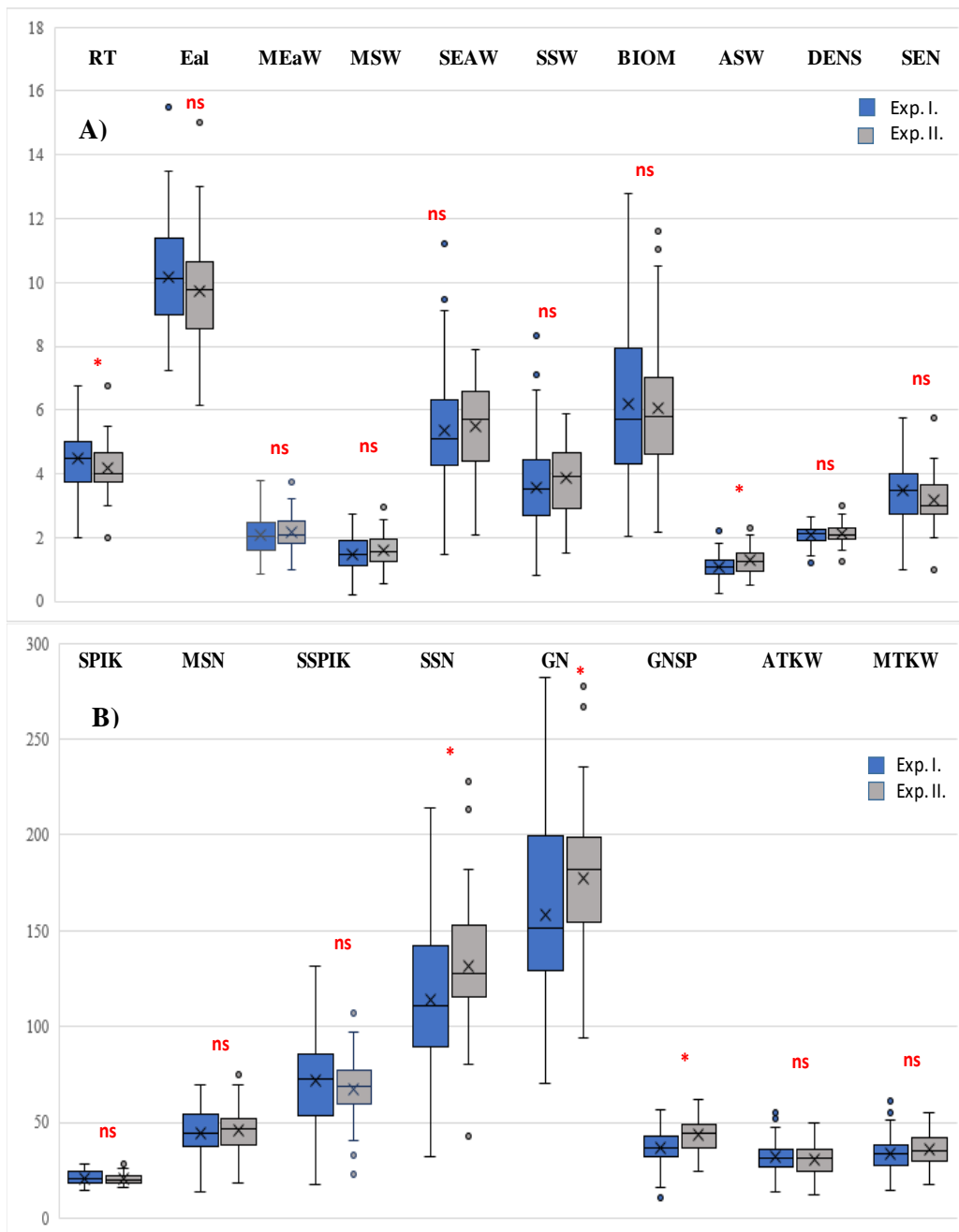

Supplement: S1 Fig — Comparison of control treatments for eighteen properties (A-B) tested in two heat stress experiments (I.-II.). A): RT—Reproductive tillers, EaL—Main ear length, MEaW—Main ear weight, MSW—Main seed weight, SEAW—Side ear weight, SSW—Side seed weight, BIOM—straw biomass, ASW—Average seed weight, DENS—Spike density (spikelet number/cm), SEN—Side ears number. B): SPIK—Spikelet number per main ear, MSN—Main seed number, SSPIK—Spikelet number per total side ears, SSN—Side seed number, GN—Grain number, GNSP—Grain number per spike, ATKW—Average thousand kernel weight, MTKW—Main thousand kernel weight; ns—not significant, *—significant at the P≤ 0.05 level. (PDF) [file pone.0252070.s001.pdf]

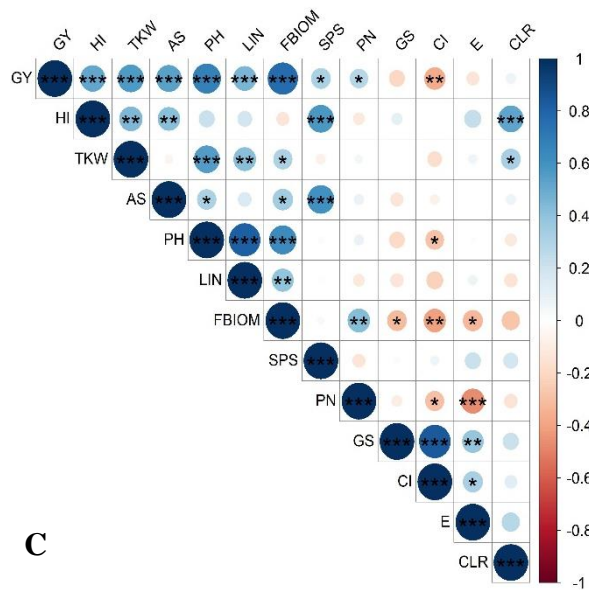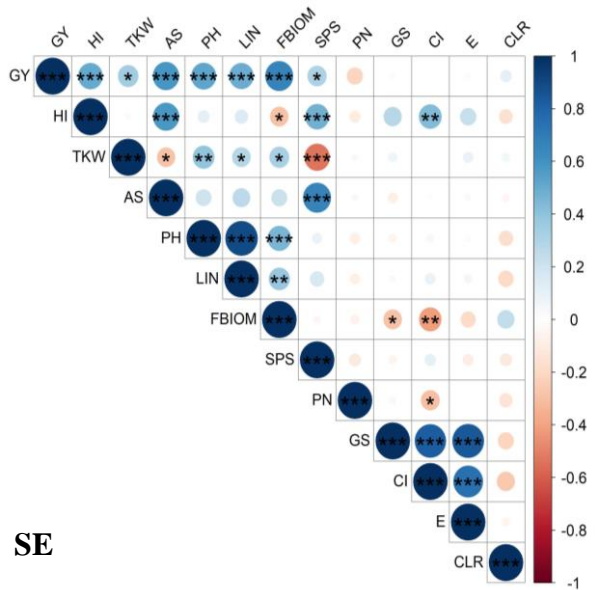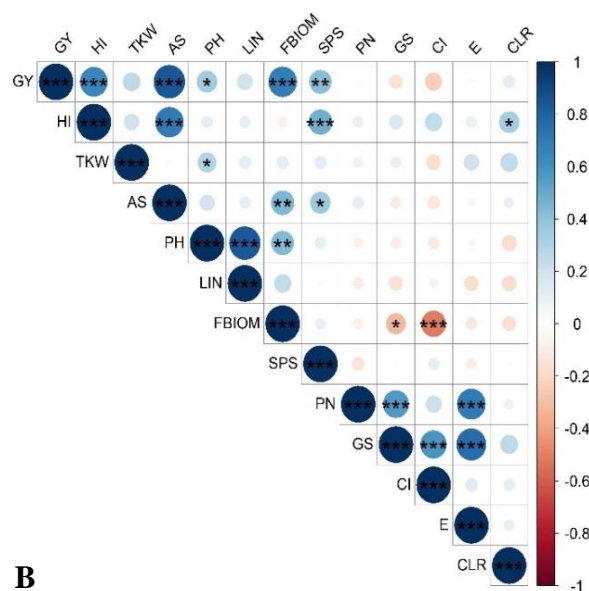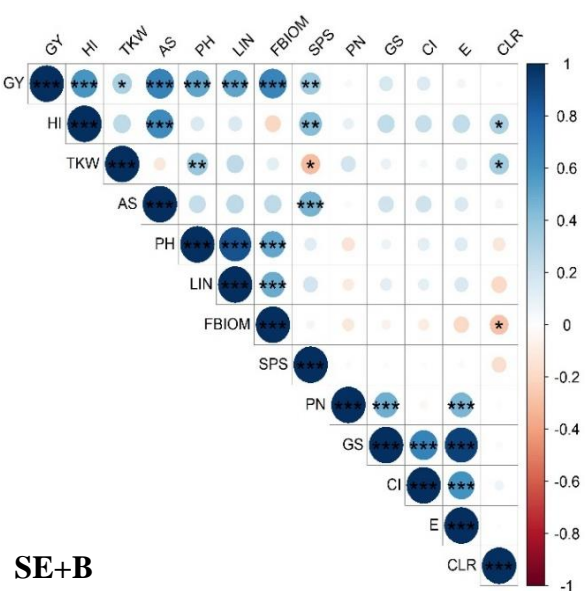

Supplement: S2 Fig — (*, **, *** critical r values of the correlation coefficient at 0.2732 P = 5%, 0.3541 P = 1%, 0.4433 P = 0.1%; n = 51); C—Control condition, SE—Single heat stress at stem elongation stage, B—Single heat stress at booting, and SE+B—Repeated heat stresses at stem elongation and booting stage; GY—Grain yield, HI—Harvest index, TKW—Thousand kernel weight, AS—Average seed number, PH—Plant height, LIN—Last internode length, FBIOM—Total aboveground biomass (straw + all ears), SPS—Grain number per spikelet, PN—Net assimilation, GS—Stomatal conductance, CI—Intercellular CO2 concentration, E—Transpiration, CLR—Chlorophyll content. (PDF) [file pone.0252070.s002.pdf]

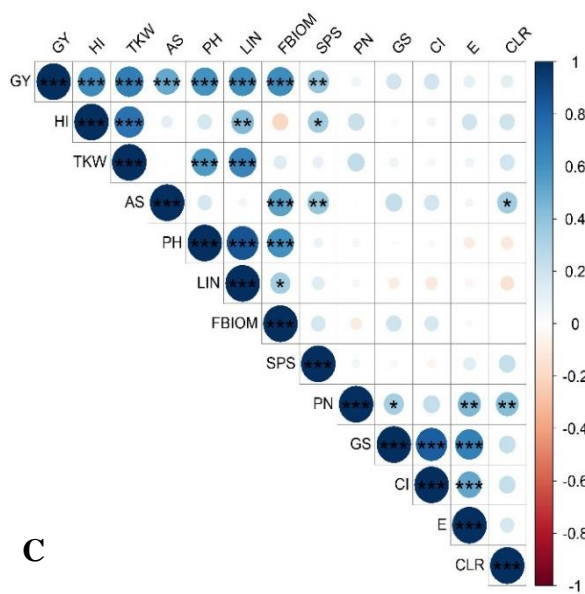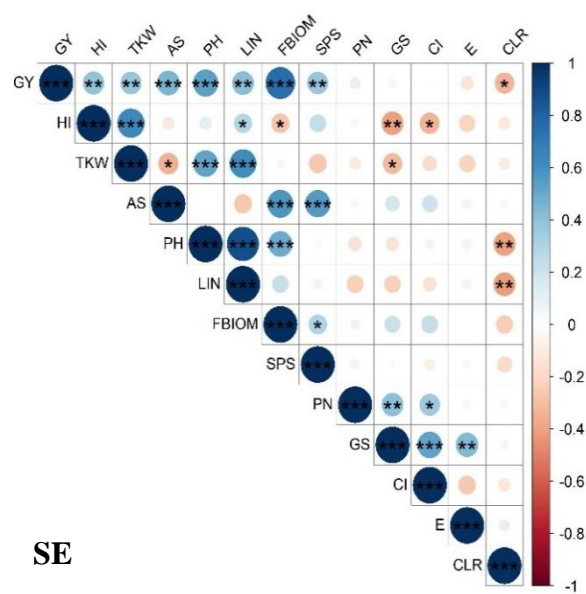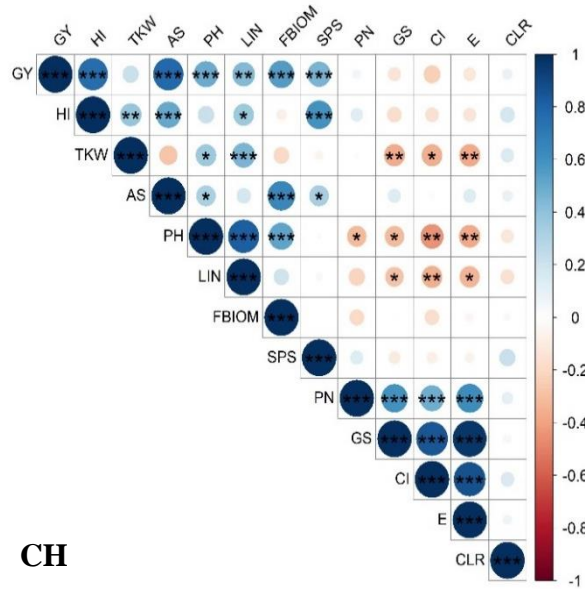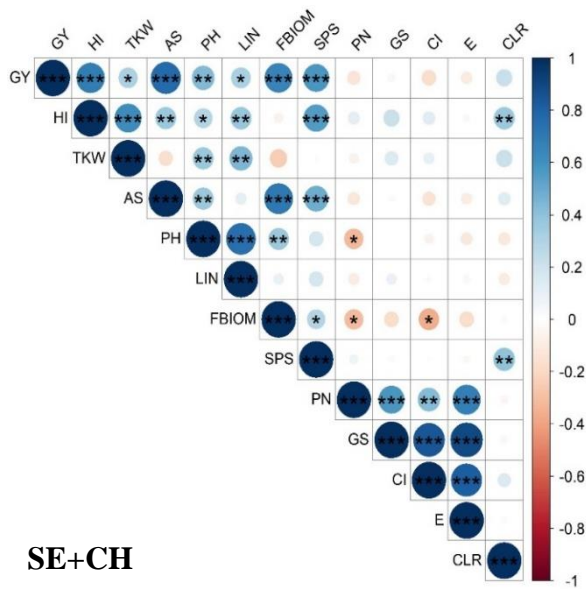

Supplement: S3 Fig — (*, **, *** critical r values of the correlation coefficient at 0.2732 P = 5%, 0.3541 P = 1%, 0.4433 P = 0.1%; n = 51); C—Control condition, SE—Single heat stress at stem elongation stage, CH—Single heat stress at heading, and SE+CH—Repeated heat stresses at stem elongation and heading; GY—Grain yield, HI—Harvest index, TKW—Thousand kernel weight, AS—Average seed number, PH—Plant height, LIN—Last internode length, FBIOM—Total aboveground biomass (straw + all ears), SPS—Grain number per spikelet, PN—Net assimilation, GS—Stomatal conductance, CI—Intercellular CO2 concentration, E—Transpiration, CLR—Chlorophyll content. (PDF) [file pone.0252070.s003.pdf]
