# Supplementary material for: Single versus repeated heat stress in wheat: What are the consequences in different developmental phases?
Source: PLoS One. 2021 May 25;16(5):e0252070. doi: 10.1371/journal.pone.0252070 (PMC8148339; doi:10.1371/journal.pone.0252070)
Supplement: S1 Table — * in Balla et al (2019) Group 1, 2, and 3 were the best yielding, Group 4, 5, and 6 were the low yielder, while Group 7 and 8 were intermediate. (PDF) [file pone.0252070.s004.pdf]

				Phenotypic cluster positions in	
	**Taxa**	**Pedigree**	**Country**	**Balla et al. (2019)***	**Present study**
**1.**	K9	DISPONENT	DE	**1**	**3**
**2.**	K194	CADENZA	GB	**1**	**7**
**3.**	K161	LUDWIG	AT	**1**	**7**
**4.**	K25	GK-HATTYU	HU	**2**	**7**
**5.**	K140	RECITAL	FR	**2**	**4**
**6.**	K64	FLEISCHMANN-481	HU	**2**	**6**
**7.**	K27	ROANE	US	**2**	**3**
**8.**	K43	LUPUS	AT	**2**	**6**
**9.**	K134	NORDIC	US	**2**	**5**
**10.**	K108	CUTTER	US	**3**	**3**
**11.**	K159	DUMBRAVA	RO	**3**	**7**
**12.**	K110	ELLVIS	DE	**3**	**3**
**13.**	K35	AURA	RO	**4**	**2**
**14.**	K6	MV-PALOTAS	HU	**4**	**2**
**15.**	K57	FENG-YOU-3	CN	**4**	**2**
**16.**	K24	LANGFANG-3	CN	**4**	**3**
**17.**	K42	COURTOT	FR	**5**	**2**
**18.**	K96	BASTIDE	FR	**5**	**5**
**19.**	K107	CHARA	AU	**5**	**2**
**20.**	K74	BUCK-PANADERO	AR	**5**	**1**
**21.**	K170	SOISSONS	FR	**5**	**4**
**22.**	K120	KLEIN-FLECHA	AR	**5**	**3**
**23.**	K185	MV-TOBORZO	HU	**5**	**1**
**24.**	K101	BLASCO	IT	**5**	**2**
**25.**	K26	MV17-09	HU	**6**	**2**
**26.**	K31	NZ4321-114	US	**6**	**2**
**27.**	K41	SALAMOUNI	US	**6**	**5**
**28.**	K188	MV-VERBUNKOS	HU	**6**	**2**
**29.**	K61	TURKMEN	TR	**7**	**1**
**30.**	K109	DIVANA	HR	**7**	**1**
**31.**	K167	SAN-PASTORE	IT	**7**	**1**
**32.**	K200	GERONIMO	IT	**7**	**4**
**33.**	K81	NW98S097	US	**7**	**1**
**34.**	K139	RAVENNA	IT	**7**	**1**
**35.**	K129	MV-AMANDA	HU	**7**	**2**
**36.**	K158	DEMETRA-OS	HR	**7**	**4**
**37.**	K141	RED-RIVER-68	US	**7**	**2**
**38.**	K157	BRIANA	RO	**7**	**4**
**39.**	K13	AGENT	US	**7**	**6**
**40.**	K17	GK-HEJA	HU	**7**	**5**
**41.**	K94	BALANCE	FR	**7**	**4**

**42.**	K47	HALLAM	US	**7**	**5**
**43.**	K114	GK-GONCOL	HU	**7**	**6**
**44.**	K15	BURATINO	CZ	**7**	**3**
**45.**	K37	MV-HOMBAR	HU	**7**	**1**
**46.**	K79	MV27-07	HU	**7**	**6**
**47.**	K93	BALADA	CZ	**8**	**6**
**48.**	K91	ALTAY-2000	TR	**8**	**5**
**49.**	K192	KWS-SCIROCCO	DE	**8**	**7**
**50.**	K160	LIBELLULA	IT	**8**	**4**
**51.**	K171	TOMMI	DE	**8**	**2**
