# Supplementary material for: Single versus repeated heat stress in wheat: What are the consequences in different developmental phases?
Source: PLoS One. 2021 May 25;16(5):e0252070. doi: 10.1371/journal.pone.0252070 (PMC8148339; doi:10.1371/journal.pone.0252070)
Supplement: S2 Table — PH—Plant height, LIN—Last internode length, EaL—Main ear length, SPIK—Spikelet number per main ear, DENS—Spike density (spikelet number/cm), SEN—Side ears number, GN—Grain number, GNSP—Grain number per spike, SSPIK—Spikelet number per total side ears, GY—Grain yield, AS—Average seed number, ASW—Average seed weight, BIOM—straw biomass, MEaW—Main ear weight, MSN—Main seed number, MSW—Main seed weight, FBIOM—Total aboveground biomass (straw + all ears), HI—Harvest index, SPS—Grain number per spikelet, SEAW—Side ear weight, SSN—Side seed number, SSW—Side seed weight, RT—Reproductive tillers, TKW—Thousand kernel weight, ATKW—Average thousand kernel weight, MTKW—Main thousand kernel weight, EVP—Evaporation, GS—Stomatal conductance, PN—Net assimilation, CI—Intercellular CO2 concentration, CLR—Chlorophyll content; ***, **, * difference significant at the 0.1%, 1% and 5% probability level. (PDF) [file pone.0252070.s005.pdf]

**Exp. I.**	Treatment (T)	Genotype (G)	T x G
**PH**	6.33***	**76.07*****	2.84***
**LIN**	20.79***	**53.74*****	7.05***
**EaL**	3.54***	**73.45*****	5.68***
**SPIK**	1.71***	**74.34*****	6.49***
**DENS**	0.43**	**64.95*****	8.09***
**SEN**	7.02***	**36.70*****	8.82***
**GN**	18.48***	**38.66*****	11.73***
**GNSP**	17.66***	**37.93*****	15.77***
**SSPIK**	1.94***	**52.22*****	6.61***
**GY**	8.46***	**45.42*****	14.07***
**AS**	13.33***	**33.05*****	18.52***
**ASW**	6.06***	**36.24*****	23.92***
**BIOM**	1.18***	**82.52*****	3.69***
**MEaW**	11.07***	**46.36*****	18.30***
**MSN**	22.77***	**37.73*****	14.19***
**MSW**	10.53***	**41.46*****	21.29***
**FBIOM**	4.97***	**69.92*****	5.25***
**HI**	4.61***	**56.69*****	13.04***
**SPS**	26.77***	**29.68*****	17.91***
**SEAW**	8.66***	**44.18*****	10.96***
**SSN**	14.26***	**36.49*****	13.26***
**SSW**	9.42***	**40.50*****	13.91***
**RT**	7.02***	**36.70*****	8.82***
**TKW**	6.71***	**46.58*****	21.13***
**ATKW**	2.29***	**43.28*****	24.18***
**MTKW**	18.93***	**36.55*****	15.02***
**EVP**	**41.55*****	4.72***	36.22***
**GS**	**40.10*****	3.22***	33.46***
**PN**	**51.71*****	5.15***	22.65***
**CI**	**28.06*****	9.65***	47.39***
**CLR**	23.42***	**24.43*****	31.36***
