# Supplementary material for: Single versus repeated heat stress in wheat: What are the consequences in different developmental phases?
Source: PLoS One. 2021 May 25;16(5):e0252070. doi: 10.1371/journal.pone.0252070 (PMC8148339; doi:10.1371/journal.pone.0252070)
Supplement: S4 Table — (PDF) [file pone.0252070.s007.pdf]

**Characteristics of K-mean clustering**	**Number of clusters checked**								
	2	3	4	5	6	**7**	8	9	10
Sum of within-cluster distances (K)	54.1	46.2	43.2	40.9	39.8	**36.8**	36.5	34.3	32.2
Difference in K between two consecutive clusters (ΔK)		7.9	2.9	2.2	1.1	**3.0**	0.3	2.1	1.8
