# Supplementary material for: Single versus repeated heat stress in wheat: What are the consequences in different developmental phases?
Source: PLoS One. 2021 May 25;16(5):e0252070. doi: 10.1371/journal.pone.0252070 (PMC8148339; doi:10.1371/journal.pone.0252070)
Supplement: Attachment — Submitted filename: Response to Reviewers.docx [file pone.0252070.s008.docx]

**PONE-D-21-06889**:

Manuscript title: **Single versus repeated heat stress in wheat: what are the consequences in different developmental phases?**

**Response to Reviewers:**

Reviewer #1: “The information provided in this paper was of good quality and will be important to the future of this area of research. However, I found the majority of the paper hard to read due to the many grammatical errors and sentences that were structured without finesse. If this paper were to be accepted, I would suggest that the authors heavily edit and focus on restructuring sentences and some paragraphs so that they use less, but more impactful words. This will make the paper much more friendly to a wide range of readers.”

Response:

We employed the professional scientific editing service, the American Journal Experts (AJE), they re-edited and revised the complete manuscript (taking care to correct grammatical errors and sentences and comply with formal requirements) according to the submission guideline of PLOS ONE. For incomprehensible sentences, we also focused on re-editing them. Below you find the Editing Certificate of the manuscript PONE-D-21-06889.
